# Novel Assessment of Urinary Albumin Excretion in Type 2 Diabetes Patients by Raman Spectroscopy

**DOI:** 10.3390/diagnostics10030141

**Published:** 2020-03-03

**Authors:** Jose L. Flores-Guerrero, Aaron Muñoz-Morales, Freddy Narea-Jimenez, Ricardo Perez-Fuentes, Enrique Torres-Rasgado, Guadalupe Ruiz-Vivanco, Naara Gonzalez-Viveros, Jorge Castro-Ramos

**Affiliations:** 1 Department of Internal Medicine, Division of Nephrology, University Medical Center Groningen, University of Groningen, 9713 GZ Groningen, The Netherlands; 2 Department of Physics, Faculty of Experimental Sciences and Technology, Center of Medical and Biotechnology Research, University of Carabobo, Valencia 2005, Venezuela; aamunoz@uc.edu.ve; 3 Optics coordination, Biomedical Optics Group, National Institute of Astrophysics, Optics and Electronics, INAOE, Puebla 72840, Mexico; fjnarea@inaoep.mx (F.N.-J.); naara@inaoep.mx (N.G.-V.); jcastro@inaoep.mx (J.C.-R.); 4 Department of Chronic Disease Physiopathology, East Center of Biomedical Research, Mexican Social Security Institute, CIBIOR, Puebla 74360, Mexico; ricardo.perezf@correo.buap.mx (R.P.-F.); guadalupe.ruizv@correo.buap.mx (G.R.-V.); 5 Faculty of Medicine, Meritorious Autonomous University of Puebla, BUAP, Puebla 72589, Mexico; enrique.torres@correo.buap.mx

**Keywords:** Raman spectroscopy, albumin, type 2 diabetes, diabetic nephropathy, kidney disease

## Abstract

Urinary albumin excretion remains the key biomarker to detect renal complications in type 2 diabetes. As diabetes epidemy increases, particularly in low-income countries, efficient and low-cost methods to measure urinary albumin are needed. In this pilot study, we evaluated the performance of Raman spectroscopy in the assessment of urinary albumin in patients with type 2 diabetes. The spectral Raman analysis of albumin was performed using artificial urine, at five concentrations of albumin and 24 h collection urine samples from ten patients with Type 2 Diabetes. The spectra were obtained after removing the background fluorescence and fitting Gaussian curves to spectral regions containing features of such metabolites. In the samples from patients with type 2 diabetes, we identified the presence of albumin in the peaks of the spectrum located at 663.07, 993.43, 1021.43, 1235.28, 1429.91 and 1633.91 cm^−1^. In artificial urine, there was an increase in the intensity of the Raman signal at 1450 cm^−1^, which corresponds to the increment of the concentrations of albumin. The highest concentration of albumin was located at 1630 cm^−1^. The capability of Raman spectroscopy for detection of small concentrations of urinary albumin suggests the feasibility of this method for the screening of type 2 diabetes renal complications.

## 1. Introduction

Type 2 diabetes (T2D) is one of the major public health problems worldwide, as its prevalence has quadrupled in the past three decades [1]. Diabetic kidney disease (DKD) is one of the main complications of T2D, and it is the leading cause of end-stage kidney disease worldwide [2]. Complications of T2D, such as DKD, imply a remarkable economic burden for the most vulnerable populations [3].

DKD is a micro vascular complication [4], which is characterized by the increment in the excretion of urine albumin, glomerular lesions, and a decrease of the estimated glomerular filtration rate (eGFR) [5,6]; it is also associated with increased mortality risk [7].

The cornerstone for the diagnosis and categorization of DKD remains the urinary albumin excretion (UAE) [8]. Microalbuminuria (UAE excretion of 30 to <300 mg/day) has been stablished as a risk marker for cardiovascular and kidney disease in several populations, and it is also associated with mortality risk. It is suggested that microalbuminuria should be monitored once or twice a year, particularly in individual with high cardiometabolic risk [9]. Therefore, microalbuminuria screening in patients with T2D is clinically relevant because its presence can modify the therapeutic approach of the patient and improve the prognosis of the renal function.

The conventional UAE measurement method is the immunochemical assay with anti-albumin antibodies. Several methods are also utilized, i.e., nephelometry, immunoturbidimetry, enzyme- linked immunosorbent assays and radioimmunoassay, and, most recently, high-performance liquid chromatography [10]. As the diabetes epidemy increases, particularly in low-income countries [11], alternative low-cost methods such as urinary dipsticks have been evaluated, exhibiting poor sensitivity and therefore a deficiency in detection [12]. Thus, new low-cost methods to measure UAE are needed.

Over the last years, there has been an important advance in the development of noninvasive biomedical optical techniques to improve and facilitate the diagnosis and screening procedures of diseases that represent a public health threat [13,14].

One promising new approach to measure UAE is Raman spectroscopy, an optical technique with potential medical applications [15,16]. This technique is based on the inelastic scattering of light by polarizable molecules that reveal the vibrational energy levels of the chemical bonds in the molecule [17,18]. Thus, Raman spectroscopy permits carrying out a rapid and nondestructive analysis of the chemical structures in biological fluids, such as cerebrospinal fluid, plasma, and urine [19]. Several studies have demonstrated the feasibility of Raman spectroscopy measurement of different biomarkers: cytochrome, amide III, and amide in saliva [20] and fibrinogen in blood [21], among others.

Such properties make Raman spectroscopy a promissory technique for low-cost and noninvasive diagnostic technology, given the fact that samples do not require any preparation before the measurement [22]; this particular feature not only reduce the cost, but it may represent a more sustainable technique, since it may reduce the use of single-use plastic laboratory consumables.

Furthermore, there is a variation of this method, denominated Surface-Enhanced Raman Spectroscopy (SERS) [23], which requires the use of silver or gold nanoparticles in order to improve the detection of the Raman spectra [24,25]. By means of SERS, it has been possible to identify the Raman spectra of albumin in bovine serum, using gold nanoparticles [26]. Likewise, the use of silver nanoparticles has permitted the detection of albumin in urinary human samples by means of SERS [27,28].

However, Raman spectroscopy has not yet been investigated as a tool to assess UAE without the enrichment of gold or silver nanoparticles. Therefore, the aim of the present study is to investigate the capability of Raman spectroscopy, to identify the presence of microalbuminuria in patients with T2D.

## 2. Materials and Methods

### 2.1. Raman Spectroscopy

Raman spectroscopy measurements were made with the Raman spectrometer QE 65000 (Oceans Optics), with a resolution of 0.14–7.7 nm FWHM (6 cm^−1^) and an integration time from 8 ms to 5 min. We selected the spectral range that varies from 200 to 1800 cm^−1^. The experimental wavelength of excitation was 785 nm, with a resolution of 2 cm^−1^. Given the fact that, in the present study, we did not add any substance (i.e., gold/silver nanoparticles) to enhance the detection of the spectra, we tested different exposure times and selected the one that provided the more defined Raman spectra: an exposure time of 60 s (5 accumulation).

A Raman probe RIP-RPB with two fibers of diameter 200 and 105 μm was used. The probe had a lens and band-pass after the output excitation fiber and dichroic filter, and this allowed it to transmit only the wavelength of laser and reflect scattering of the sample. A focal length of 7.5 mm was used. All spectra were obtained at a working distance of 4.8 mm. Human and artificial samples were placed in wells containing 5 mL of the sample. The experiments were conducted according to the American National Standard for safe use of lasers (ANZI Z136.1)

### 2.2. Fluorescence Background Removal

Given the fact that the background fluorescence in Raman spectroscopy affects the accuracy signal detection, background fluorescence was removed. In order to remove the fluorescence background in the Raman spectrum, the method described by Villanueva-Luna et al. [29] was used. The technique is based in wavelets theory, using symlets and bi-orthogonals wavelets, which improve the accuracy in the determination of spectral peaks. This method has previously been tested in Raman spectra ranging from 300 to 1800 (cm^−1^), of different biological samples, excited with a 785 nm laser. The spectrum was broadened, and the profile of the set of all spectral lines was enveloped and fitted by a gaussian or Lorentz function. To depict the composition of the different analytes on patients, the Raman-active spectral regions were identified by applying a fluorescence removal method [30] and fitted Gaussian curves to spectral regions containing features of creatinine, water, urea, and albumin.

### 2.3. Synthetic Urine

In order to observe the Raman spectra of the different concentrations of albumin in the synthetic urine, two types of solutions were prepared. The first type of solution was prepared with sterile water for injection and various concentrations of albumin in a 5 mL total volume of dissolution. Concentrations of albumin were 0.040, 0.060, 0.080, 0.100, and 0.200 (mg/mL).

The second type of solution (artificial urine) was prepared in order to emulate the composition of urine. The artificial urine was composed of human albumin (MP Biomedicals, Irvine, CA, USA), urea (04821530 MP Biomedicals, USA), creatinine (C4255-100G Sigma-Aldrich, St. Louis, MO, USA) diluted in injectable water, and different concentrations of albumin. Six different solutions of artificial urine were prepared, concentrations of urea were constant (0.05 g), as well as creatinine (3.35 g); concentrations of albumin were gradually increased from 0.0 to 0.1 g.

The measurement error in relation to the concentration of albumin was calculated through the first derivate, given the fact that the concentrations used in the experiments only involved 2 variables (mass/volume). The electronic balance used in the experiments may present an error of 0.01 mg, and the employed pipette could present an error of 0.02 mL. The percentage of concentration (P_c_) could be calculate by using the mass of solute (m_s_), and the total volume (V_t_) of the solution P_c_ = (m_s_/V_t_). The error in the percentage concentration depends on the uncertainty of the pipette by which the volume was measured and the scale with which we measured the mass. (δPc) = (V_t_ δ m_s_ − m_s_ δ V_t_)/(V_t_^2^). The reported error in the measurement of the utilized spectrometer for each of the peaks is 10 cm^−1^. Samples of synthetic urine did not receive any treatment or enrichment before the measurement.

### 2.4. Human Urine

Urinary samples were taken from a 24 h urine collection of ten patients with a confirmed diagnosis of T2D attending the outpatient clinic of the Mexican Social Security Institute Clinic 2. Pregnant women, subjects with type 1 diabetes, or patients with any underlying autoimmune disease were excluded. The urine collection was performed according to the standard procedure of the 24 h Urine Study Procedures Manual of the Centres for Disease Control and Prevention (CDC). Samples of human urine did not receive any treatment or enrichment before the measurement.

The protocol for the present study was approved by the local ethics committee of the Mexican Social Security Institute. All participants in the present analysis provided written informed consent to participate, and all study procedures were conducted according to the Declaration of Helsinki.

## 3. Results

### 3.1. Raman Spectrum of the Components of Artificial Urine

**Figure 1:**
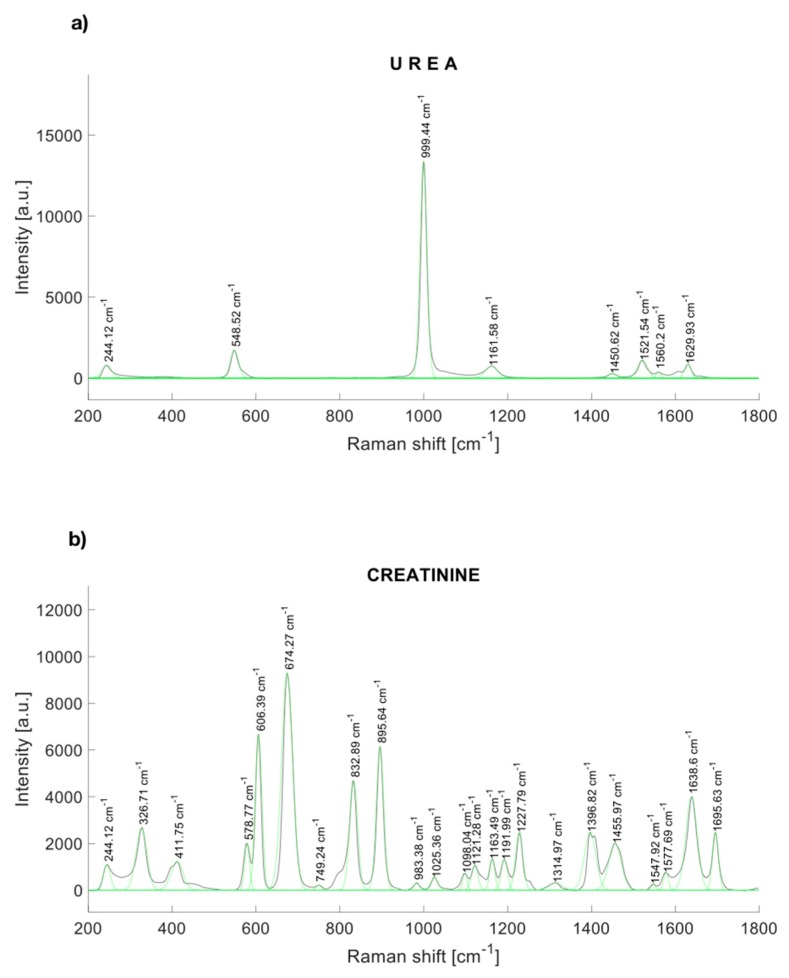
Raman spectrum of (**a**) urea, and (**b**) synthetic creatinine.

The characteristic peaks of urea are localized at 244.12, 548.52, 999.44, 1161.58, 1448.54, 1521,54, and 1629.93 cm^−1^ [30] (Figure 1a). Figure 1b shows the Raman response of synthetic creatinine (C4255-100G-SIGMA, Cleveland, OH, USA) diluted in water. The main Raman molecular vibrations are in the range between the Raman displacements from 550 to 940 cm^−1^, although other spectral bands are notorious. The principal Raman peaks of creatinine were found near to 327, 420, 576, 598, 606, 681 [31], 846 [31], 908 [31], 1224, 1396.82, 1469, 1638, and 1690 cm^−1^.

**Figure 2:**
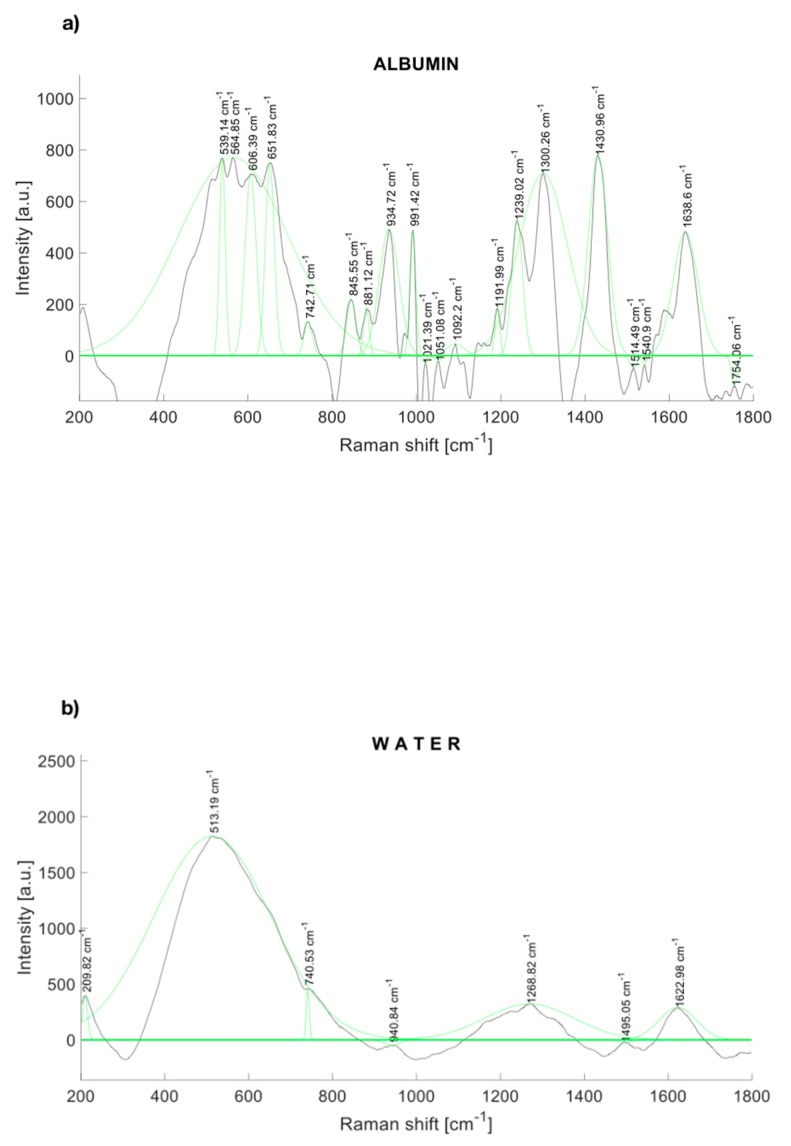
Raman spectrum of (**a**) albumin and (**b**) water, with a fitting Gaussian curve applied.

The Raman spectra of human albumin (Albumin (Human); MP Biomedicals, Irvine, CA, USA) are depicted in Figure 2a. Albumin was identified within several significant Raman bands near to 560, 672 [32], 709 [32], 775 [32], 842 [32], 940 [33], 960 [33], 1002 [33], 1089 [33], 1102 [33], 1157 [33], 1172 [33], 1319 [33], 1420 [34], 1443 [33], 1650 [35], and 1750 cm^−1^.

Likewise, Figure 2b depicts the Raman spectrum of water, which represents 95% of the urine. Water also contributes to Raman peaks near to 277.98, 396.57, 416.67, 514.13, 790.3 [36], 1203, 1349, 1533, 1641 [36], 1656 [34], and 2167 [34] cm^−1^.

**Figure 3:**
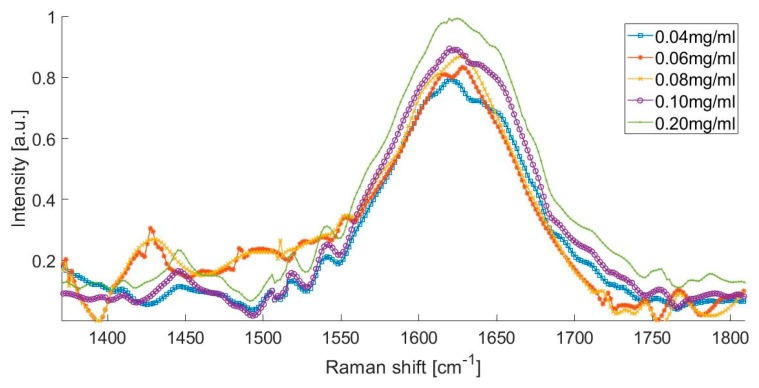
Raman spectrum of different albumin concentrations.

The Raman spectra of the artificial urine sample with different albumin concentrations, from 0.04 to 0.2 mg/mL is presented in Figure 3. This spectrum shows that the presence of urea peaks, creatinine peaks, water peaks, and the formation of a small peak in the spectral range from 1400 to 1500 cm^−1^, which could be attributed to the presence of albumin in the urine.

The Raman spectrum of the artificial urine sample with concentrations of 0.080 and 0.100 mg/mL was within the range of the microalbuminuria of the laboratory tests (Figure 3). There was an increase in the intensity of the Raman signal at 1450 cm^−1^, which corresponds to the increment in the concentration of albumin in the solution. Figure 3 shows that the Raman spectrum of the solution with the higher concentration of albumin (0.200 mg/mL) is represented with the peak observed at 1630 cm^−1^.

### 3.2. Raman Spectrum of the Components of Human Urine

**Figure 4:**
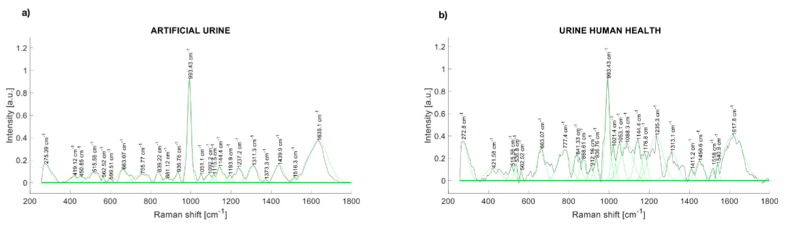
(**a**) Raman spectrum of artificial urine (water + urea + creatinine); (**b**) Raman spectrum of urinary sample (healthy control).

The comparison of the Raman spectra of the artificial urine and human urine are presented in Figure 4. The Raman peaks of synthetic human urine were found at 514, 662, 836, 937, 993, 1143, 1239, 1307, 1433, and 1631 cm^−1^ (Figure 4a). The main Raman peaks of a health participant urine sample are at 280.57, 536.8, 663.07, 777.4, 841.33, 936.76, 993.43, 1053.1, 1088.3, 1144.4, 1235.3, 1313.1, 1450.6, and 1617.8 cm^−1^ (Figure 4b).

The concentrations of urea and creatinine established in Table 1 were identified in the spectra of urinary samples (total volume 5 ml). Such solutions were characterized by peaks in the spectral region from 500 to 560 cm^−1^, from 960 to 1043 cm^−1^, and from 1120 to 1192 cm^−1^ for urea; the spectral range from 650 to 940 cm^−1^ was used to characterize creatinine, and from 1550 to 1750 cm^−1^ corresponded to water.

**Figure 5:**
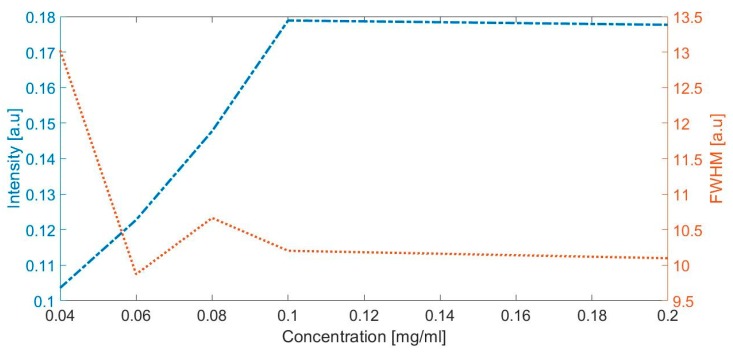
The intensity and full width at half maximum (FWHM) vs. albumin concentration.

The highest concentration of albumin (clinical albuminuria) represented with a peak observed in 1630 cm^−1^ (Figure 3) was evaluated with the intensity and full width at half maximum. Figure 5 depicts the intensity and full width at half maximum (FWHM) vs. albumin concentration; the lines represent the trend of intensity and the average width of the specific peaks at the Raman shift in 1443 cm^−1^, when the concentration of albumin is increased in the artificial urine samples.

**Figure 6:**
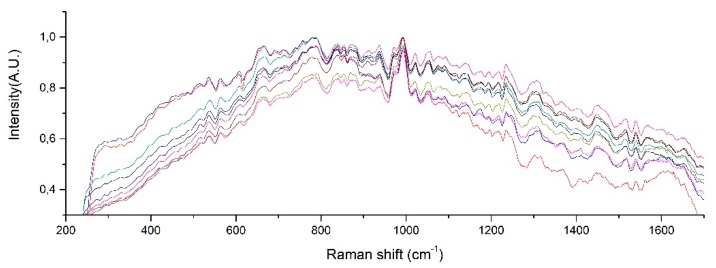
Raman peaks of human urine samples of 10 patients with T2D.

**Figure 7:**
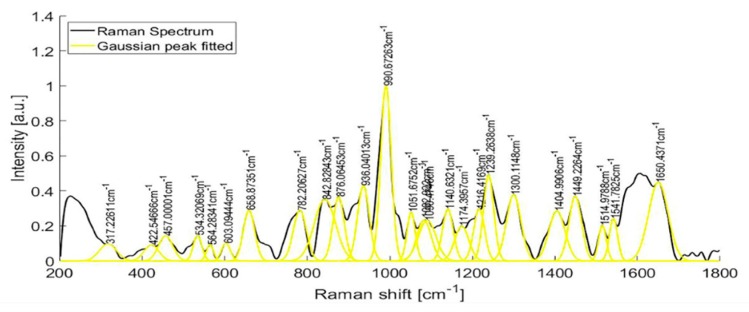
Raman spectra of the urine sample of a patient with T2D, before and after fitting a Gaussian-line shape.

Raman spectra from samples of ten patients with T2D are shown in Figure 6. After fitting a Gaussian-line shape to the Raman spectrum, and by taking account of the width, the area under the curve, and the maximum intensity, we found the most notable Raman peaks of human urine at 421.58, 450.85, 515.56, 536.8, 582.52, 608.68, 663.07, 686.11, 757.94, 779.55, 839.22, 853.95, 868.61, 910.1, 936.75, 993.43, 1021.39, 1055.01, 1090.25, 1154.36, 1195.77, and 1235.28 cm^−1^. Raman peaks of urinary samples of patients with T2D were located at 534.44, 654.08, 779.55, 837.11, 936.76, 993.43, 1055.01, 1142.44, 1235.28, 1447.05, 1517.03, 1539.11, 1616.08, and 1887.21 cm^−1^. The use of the background removal method, which was previously developed by our group [29], shows an improvement in the definition of the Raman peaks (Figure 7). Few of those peaks correspond to any molecular vibration. The peaks in the urine spectrum which show the presence of albumin are located at 663.07 [37], 993.43 [37], 1021.43, and 1235.28 [37] cm^−1^. The reported FWHM (cm^−1^) and tentative band assignment are summarized in Table 2; it is noteworthy that the peal 660.09 cm^−1^ is the most marked peak showed in Figure 2.

## 4. Discussion

In this present study, we investigated for first time the feasibility of Raman spectroscopy to identify the presence of albumin in human urine. Based on the results obtained, Raman spectroscopy is useful to determine the presence of albumin in artificial urine samples, and it could be used as a control tool in patients with T2D. When analyzing the Raman spectra obtained from artificial urine by increasing the concentration of albumin, we found that an increased intensity at the peak of 1450 cm^−1^ may correspond to amino acid glycine concentration in albumin, representing a Raman window for the detection of the presence and concentration of the albumin in the urine.

In order to confirm these results, we presented the direct proportionality between the intensity of peak and the concentration of albumin (Figure 5), and our results are agreed with those obtained by Lin et al. [38]. Lin and collaborators reported that the Raman response to albumin concentrations in plasma is assigned this response in 1448 cm^−1^ to the amino acid glycine, which is one of the 585 amino acids with disulfide bridges crosslinked in albumin.

Albumin has two peaks identified as 1431 cm^−1^ and 1633 cm^−1^, as can be seen in Figure 2a. Water presents an intense peak in the range from 1500 to 1700 cm^−1^ at 1623 cm^−1^, as shown in Figure 2b. We made several solutions with different concentrations (Table 1). The effects of those albumin concentrations in the Raman spectrum are shown in Figure 1. When albumin was dissolved in sterile water, one peak overlapped the Raman signal of water at 1630 cm^−1^; nonetheless, the mix of water with albumin presents another detection peak at 1431 cm^−1^, which reinforces the idea that Raman spectroscopy can be a useful tool to detect albumin in water dissolutions. The shape of the Raman seems to be in line with the previous report of the SERS technique where nanoparticles were added to the urinary sample before the measurement [28]. In such work, urinary albumin in a sample with concentration of 10 μg/mL presented a double Raman peak at 1002−1026 cm^−1^. Nonetheless, the SERS spectra of augmenting albumin concentration samples display a peak at 1450 cm^−1^, which is close to the peak we reported, 1431 cm^−1^.

The present study has several strengths. According to the results shown in Figure 3 and Figure 4, Raman spectroscopy is shown as a potential technique to detect the presence of albumin in the urine. Although Raman spectroscopy has been used to detect changes in the concentrations of compounds like urea, creatinine, and glucose in the urine [30,31], it has not been used for the determination of microalbuminuria, which is one of the most import biomarkers for kidney disease in the early phase that would allow taking timely clinical actions to avoid major complication in patients with T2D, such as DKD. Moreover, Raman spectroscopy could be applied without the use of additional chemical reagents, which could reduce the costs associated with the assessment of microalbuminuria in the follow-up of patients with T2D. Furthermore, our results were not affected by the pH of the urine samples. It has been described that pH could affect the albumin concentrations after prolonged frozen storage and when the urine samples have a pH <5 [39]; the reported measurements were conducted on the same day of the urinary collection, and the mean pH of the samples was 6.04 (SD = 0.53); therefore, we avoided the potential effect of pH on the albumin concentrations.

Likewise, several limitations of the present study need to be addressed. For instance, in the present study, we did not asses the spectra of the electrolytes (Cl, Na, and F). Cl and Na present peaks in their Raman spectra over 2000 cm^−1^ [37]; in addition, F has a Raman spectrum in the range of the studied spectral of albumin. Whether the disturbances of urinary electrolytes can affect the performance of Raman spectroscopy assessment of urinary albumin is still unknown. Furthermore, whether the implementation of a combined approach, using the methodology described in this pilot study and enriched methods (i.e., with magnetic microspheres) [24], may improve the sensibility and specificity of Raman spectroscopy is unknown; therefore, further research is needed.

## 5. Conclusions

In the present study, we evaluated the performance of Raman spectroscopy as a tool to assess UAE without the enrichment of gold or silver nanoparticles. By means of prolonged exposure and fluorescence background removal using a wavelets-based method, we found that there is an increased intensity at the peak of 1450 cm^−1^, in samples with increased concentrations of albumin. The results were consistent in both artificial and human urinary samples.

The results obtained from this pilot study suggested that analytes such as creatinine, urea, water, and albumin in urinary samples from patients with T2D can be identified by Raman spectroscopy by finding their corresponding Raman peaks.

## Figures and Tables

**Table 1:** Concentrations of artificial urine components.

Sample Name	Urea	Creatinine	Albumin
Normal 1	0.05 (g)	3.35 (mg)	0.000 (mg)
Normal 2	0.05 (g)	3.35 (mg)	0.040 (mg)
Normal 3	0.05 (g)	3.35 (mg)	0.060 (mg)
Microalbuminuria Case 2	0.05 (g)	3.35 (mg)	0.080 (mg)
Microalbuminuria Case 3	0.05 (g)	3.35 (mg)	0.100 (mg)
Clinical Albuminuria	0.05 (g)	3.35 (mg)	0.200 (mg)

**Table 2:** Relevant peaks of albumin Raman spectra.

Wavenumber (cm^−1^)	FWHM (cm^−1^)	Tentative Band Assignment
660.09	55.49	*v(CS)*
841.36	36.37	*Tyr*
939.84	33.61	*V(CCN)sym, v(CC)*
993.57	26.48	*Phe*
1316.88	40.32	*δ(CH)*
1429.91	46.73	*δ(CH2)*
1633.91	41.58	*Tyr*
1762.32	36.37	Unknown
